# Dirac Point in the Charge Compensated Single-Crystal Ru_3_Sn_7_

**DOI:** 10.3390/ma18174044

**Published:** 2025-08-29

**Authors:** Xiaoyu Ji, Xuebo Zhou, Shilin Zhu, Fengcai Ma, Gang Li, Wei Wu

**Affiliations:** 1 Department of Physics, Liaoning University, Shenyang 110036, China; 2 Institute of Physics, Chinese Academy of Sciences, Beijing 100190, China

**Keywords:** Dirac point, topological material, quantum oscillations

## Abstract

Ru_3_Sn_7_ crystallizes in the cubic Ir_3_Ge_7_-type structure (space group *Im3m*), a class of intermetallic compounds. Previous studies focused primarily on its crystal structure, band calculations, and basic transport properties. Here, we report a systematic investigation of high-quality single crystals via electrical resistivity, Hall effect, specific heat, and thermal transport measurements. The T_3_X_7_ intermetallic family—with its diverse electronic ground states—provides an ideal platform for exploring such topology–property relationships. Ru_3_Sn_7_ exhibits metallic behavior, with consistent Hall effect and Seebeck coefficient data indicating a compensated electron-hole two-band system. Temperature-dependent modulation of electronic states near the Fermi surface alters charge carrier transport, which may imply the presence of a Lifshitz transition in Ru_3_Sn_7_. More importantly, magnetic quantum oscillations are observed for the first time, confirming the presence of two Dirac points in its band structure.

## 1. Introduction

The intense search for topological semimetals within condensed matter physics is fueled by their unique properties, primarily the emergence of exotic, relativistic-like quasiparticles such as massless Dirac and Weyl fermions within their bulk electronic structure. These materials hold significant promise not only for fundamental physics discoveries—offering novel platforms to explore quantum anomalies, unconventional superconductivity, and emergent quantum phases—but also for potential revolutionary applications. Their ultra-high carrier mobility, chiral magnetotransport, and robust surface states make them prime candidates for next-generation low-power electronics, quantum sensors, spintronic devices, and potentially fault-tolerant topological quantum computing architectures, promising breakthroughs in energy efficiency, high integration density, and computational power.

Among the diverse classes of topological semimetals, Dirac semimetals (DSMs) occupy a pivotal position. They host three-dimensional bulk Dirac cones—linear band crossings protected by specific crystal symmetries (such as rotational symmetry or inversion symmetry combined with time-reversal symmetry) rather than just time-reversal symmetry alone. This symmetry protection gives rise to extraordinary transport phenomena, including ultrahigh carrier mobility, giant magnetoresistance (MR) that scales non-saturatingly with a magnetic field, and the chiral anomaly under parallel electric and magnetic fields. Materials like Na_3_Bi [1] and Cd_3_As_2_ [2] have served as seminal examples of DSMs, validating theoretical predictions and showcasing these remarkable properties.

The T_3_X_7_ family of intermetallic compounds (where T is typically a transition metal like Nb, Ta, Mo, Re, Ru, and Ir, and X is a p-block element like Si, Ge, Sn, Sb, As, and Te) has emerged as a fertile and versatile ground for exploring novel quantum states and transport phenomena [3]. Crystallizing in the cubic Ir_3_Ge_7_-type structure (space group *Im3m*), this family exhibits a remarkable diversity of electronic ground states. Examples range from superconductivity (e.g., Ir_3_Ge_7_ below *T_c_* ≈ 0.87 K, Mo_3_Sb_7_ below *T_c_* ≈ 2.2 K), charge density waves (e.g., Nb_3_Sn_2_Te_5_, Ta_3_Sn_2_Te_5_), to potential topological phases (e.g., Re_3_As_7_, Nb_3_SbTe_7_) [3,4,5,6,7,8]. This inherent tunability and richness make the T_3_X_7_ family an ideal platform for investigating the intricate interplay between crystal structure, electronic correlations, and topology–property relationships.

Within this family, Ru_3_Sn_7_ stands out as a highly promising candidate predicted to host non-trivial topological electronic states. Structurally isotypic to Ir_3_Ge_7_, it features a complex cubic lattice built from Ru and Sn atoms conducive to itinerant electron behavior, a prerequisite for observing pronounced topological transport effects. Initial characterization studies primarily focused on confirming its crystal structure and basic electronic properties, often leaning towards classifying it as a conventional diamagnetic metal, especially when contrasted with its superconducting counterpart Ir_3_Ge_7_ [9]. However, this initial perspective potentially overlooked Ru_3_Sn_7′_s deeper significance. Crucially, recent theoretical advances, leveraging powerful frameworks like topological quantum chemistry (TQC) that classify materials based on symmetric eigenvalues and band representations, have strongly hinted at the existence of symmetry-protected non-trivial band crossings very near the Fermi level in Ru_3_Sn_7_. TQC analysis suggests the presence of essential band crossings characteristic of Dirac semimetals within its electronic structure.

Despite these intriguing theoretical predictions and the established structural knowledge, a significant gap persists. Prior experimental work on Ru_3_Sn_7_ has been largely limited to structural characterization and rudimentary transport measurements [9,10,11,12]. A comprehensive experimental investigation systematically linking its inherent crystal symmetry to emergent topological physics through detailed quantum transport measurements has been notably lacking. Furthermore, the synthesis and characterization of high-quality single crystals—essential for probing subtle quantum effects like magnetic quantum oscillations—have been challenging for this compound.

The present study directly addresses this critical gap. We bridge the divide between prior structural knowledge and emerging topological predictions by presenting a systematic and thorough examination of the physical properties of high-quality single crystals of Ru_3_Sn_7_. Utilizing a suite of advanced experimental techniques (electrical resistivity, Hall effect, specific heat, Seebeck coefficient, thermal conductivity, and magnetization). Notably, the synthesis of large, high-purity crystals via a carefully optimized Sn flux method (demonstrated by a high residual resistivity ratio, RRR ≈ 46) was crucial for the success of this study, enabling the first observation of magnetic quantum oscillations in this compound—a definitive probe of its Fermi surface topology and Dirac points. This work not only confirms the theoretical predictions but also establishes Ru_3_Sn_7_ as the nontrivial metal within the rich T_3_X_7_ phase space.

## 2. Experimental Methods

High-quality single crystals of Ru_3_Sn_7_ were synthesized employing the Sn flux growth technique, leveraging the significant solubility difference of Ru in molten Sn versus solid Ru_3_Sn_7_. The flux method is currently one of the most commonly used and highly effective techniques for growing single crystals in laboratory settings. The basic principle of this method involves dissolving the raw materials required for crystal growth in a low-melting-point elemental or compound solvent at high temperature. As the solution cools, the solute becomes supersaturated, leading to the precipitation of single crystals. Precursor materials consisted of high-purity Ru powder (99.95%) and Sn powder (99.999%), combined in an atomic ratio of Ru:Sn = 1:10. This deliberate excess of Sn (total mass 1–2 g) served the dual purpose of reactant and self-flux medium, promoting dissolution at high temperatures and subsequent crystallization upon cooling. The mixture was loaded into a high-purity alumina crucible (6 mL volume, cylindrical geometry: diameter φ16 mm, height 55 mm, wall thickness 1.5 mm), chosen for its chemical inertness towards molten Sn under the growth conditions. As confirmed by the Ru–Sn binary phase diagram, this specific composition in excess Sn thermodynamically favors the exclusive formation of the Ru_3_Sn_7_ phase.

The loaded crucible was subsequently sealed under dynamic high vacuum (<10^−4^ mbar) within a fused silica ampoule to prevent oxidation and Sn volatilization. The sealed assembly was then subjected to a precisely optimized three-stage thermal protocol within a programmable tube furnace:

Homogenization and Reaction: The temperature was gradually ramped (typically at 2–5 °C/min) to 1150 °C and held for an extended period of 100 h. This stage ensured complete dissolution of the Ru powder and thorough homogenization of the molten flux, facilitating the formation of the Ru_3_Sn_7_ precursor compounds.

Nucleation Dwell: Following homogenization, the temperature was maintained at 1150 °C for an additional 10 h. This dwell period is critical for establishing precise thermal equilibrium throughout the melt and initiating stable crystal nucleation sites.

Controlled Crystal Growth: The crucial crystallization stage involved a slow, linear cooling ramp from 1150 °C down to 600 °C at a rigorously controlled rate of 5 °C/hour. This slow cooling rate is essential to promote diffusion-controlled growth of large, well-faceted single crystals while suppressing the formation of secondary phases or polycrystalline aggregates. Upon reaching 600 °C, the ampoule was promptly inverted and centrifuged (≈3000 rpm for 5–10 min) to rapidly separate the molten Sn flux from the solidified Ru_3_Sn_7_ crystals trapped within the crucible.

Post-Growth Processing: Residual Sn flux adhering to the crystal surfaces was selectively removed by etching in concentrated hydrochloric acid (HCl, 37%) for several hours. This process effectively dissolves the Sn without significantly attacking the Ru_3_Sn_7_ crystals, preserving their structural integrity and surface quality. The resulting high-quality, flux-free single crystals were then rinsed thoroughly with deionized water and ethanol before drying. This synthesis route consistently yielded crystals suitable for detailed crystallographic and physical property investigations.

### 2.1. Structural Characterization

#### 2.1.1. Powder X-Ray Diffraction (PXRD)

To confirm the phase purity and bulk crystal structure, representative Ru_3_Sn_7_ single crystals were gently ground into a fine powder using an agate mortar and pestle (agate minimizes contamination). PXRD measurements were performed at room temperature using a benchtop Shimadzu XRD-7000 diffractometer (Shimadzu, Kyoto, Japan) operating in Bragg–Brentano θ–2θ geometry. The instrument was equipped with a Cu Kα X-ray source (λ = 1.540593 Å, generated from a silver anode tube), a Ni Kβ filter to suppress unwanted Kβ radiation, and a scintillation counter detector. Data were collected over a 2θ range of 10° to 80° with a step size of 0.02° and a counting time of 1–2 seconds per step.

#### 2.1.2. Single-Crystal X-Ray Diffraction (SCXRD)

For detailed structural refinement, crystals of appropriate morphology and size (typically ≈ 1 mm in largest dimension) were carefully selected under an optical microscope from the crushed sample and mounted on glass capillaries or MiTeGen micromounts. High-resolution SCXRD data collection was conducted at 273 K (0 °C) using a Bruker D8 Venture diffractometer (Bruker, Karlsruhe, Germany) equipped with a PHOTON III CPAD detector (Bruker, Karlsruhe, Germany). Mo Kα radiation (λ = 0.71073 Å) was generated by a microfocus Mo-target source with MX optics (Incoatec IµS 3.0) for high flux and beam collimation. A full sphere of reciprocal space was collected using combined φ and ω scans with a frame width of 0.3°. Data Processing: The collected reflection intensities were integrated and corrected for Lorentz, polarization, and absorption effects (empirical multi-scan method using SADABS (SADABS-2016/2)) using the Bruker APEX 3 software suite (APEX 3). Unit cell parameters were determined from the centroids of a sufficient number of high-angle reflections. Subsequent structure solutions by direct methods and refinement by full-matrix least-squares on F^2^ were performed using the SHELXTL package (SHELXL-2019/3) within APEX 3.

### 2.2. Physical Property Measurements

#### 2.2.1. Electrical Resistivity

The in-plane electrical resistivity ρ(T) was measured on selected single crystals using the standard four-probe alternating current (AC) technique to eliminate contact resistance contributions. Fine platinum (Pt) wires (typically 25 μm diameter) were attached to freshly cleaved or etched crystal surfaces using conductive silver epoxy (Dupont 4922), forming low-resistance ohmic contacts. Contact alignment and spacing were verified under a microscope. Measurements were performed in a Quantum Design Physical Property Measurement System (PPMS, model 9T) equipped with a lock-in amplifier (Stanford Research Systems SR830), typically applying a small AC excitation current (5000 μA) to avoid Joule heating. Data were acquired over a temperature range from 2 K to 300 K, often in both zero field and applied magnetic fields.

#### 2.2.2. Specific Heat

The specific heat capacity C_P_(T) of a Ru_3_Sn_7_ single crystal was measured using the thermal relaxation method implemented in the Quantum Design PPMS (9T model). The crystal was securely mounted onto a pre-calibrated, high-thermal-conductivity sapphire disc platform using a minute amount of Apiezon N grease to ensure optimal thermal contact. A calibrated Cernox™ resistance thermometer (Quantum Design, San Diego, CA, USA) was used for precise temperature sensing. Measurements were typically performed over the temperature range 2 K to 300 K in zero magnetic field.

#### 2.2.3. Magnetization

The DC magnetization M(T, H) was measured using a Quantum Design Magnetic Property Measurement System (MPMS3) (Quantum Design, San Diego, CA, USA), specifically a 7 T Superconducting Quantum Interference Device (SQUID)-based Vibrating Sample Magnetometer (VSM) (Quantum Design, San Diego, CA, USA). Single crystals were carefully mounted on a plastic straw or quartz rod sample holder. Measurements included the following:

Temperature-dependent magnetization M(T): Collected under both zero-field-cooled (ZFC) and field-cooled (FC) protocols at various applied fields (e.g., 0.1 T, 1 T) typically from 1.8 K to 300 K.

Field-dependent magnetization M(H): Isothermal magnetization loops measured at selected temperatures up to the maximum field of 7 T.

## 3. Results and Discussion

**Figure 1:**
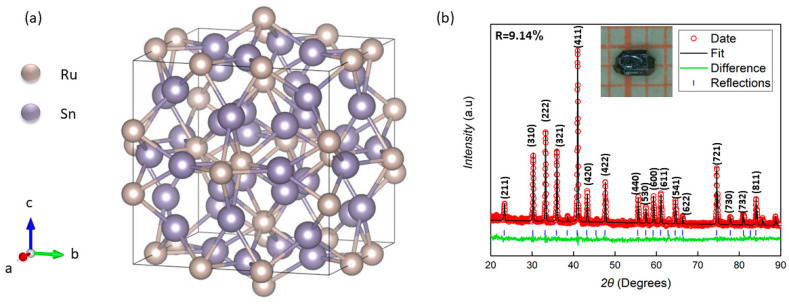
(**a**) Crystal structure of Ru_3_Sn_7_. The golden and purple spheres represent Ru and Sn atoms, respectively. (**b**) XRD pattern of powder obtained by grinding Ru_3_Sn_7_ crystals at room-temperature, with the corresponding Rietveld refinement shown as a solid line.

Figure 1a depicts the structural arrangement of the Ru-site and Sn-site atoms in the unit cell. The near-neighbor Sn atoms are joined, which defines polyhedral cavities consisting of cubes, square anti-prisms, and tetrahedra. The Ru atoms occupy the square anti-prism. Single crystals of Ru_3_Sn_7_, exhibiting a shiny appearance and well-defined facets, were successfully synthesized, with sizes reaching up to several millimeters. These crystals predominantly displayed cubic or rectangular morphologies, although a subset exhibited elongation along the <001> direction. All observed diffraction peaks were accurately indexed to the crystal planes of Ru_3_Sn_7_, as confirmed by the reference PDF card (No. 97–015-0763). A representative optical image of a typical crystal is provided in the inset of Figure 1b. Structural analysis via single-crystal X-ray diffraction revealed lattice parameters of a = b = c = 9.342 Å, which are in close agreement with previously reported values, albeit slightly lower than the a = 9.351 Å documented in the referenced literature [10]. Inevitably, the sample was mixed with some tiny impurities during its growth, so Rietveld analysis (FullProf Suite) yielded R = 9.14%, still confirming phase purity.

**Figure 2:**
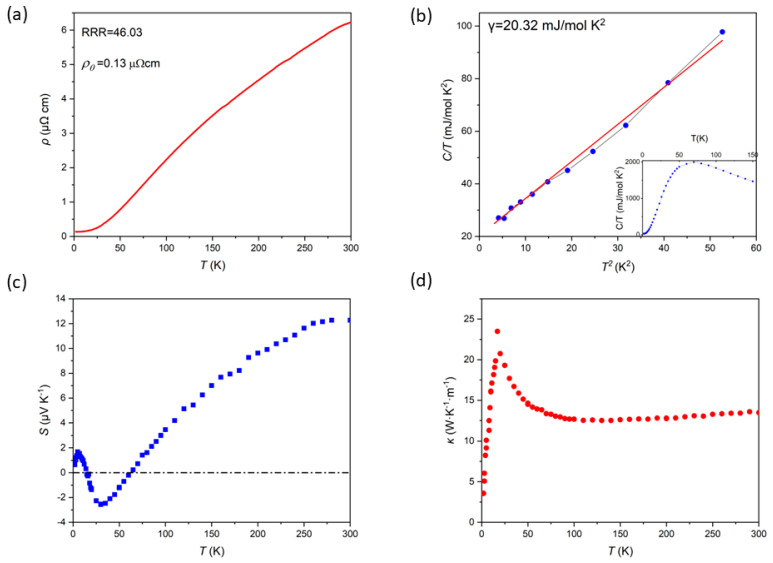
(**a**) Resistivity vs. temperature of a Ru_3_Sn_7_ single crystal. (**b**) Temperature dependence of the specific heat C_P_(T)/T of Ru_3_Sn_7_ single crystal (The blue dotted line is the actual measurement curve, the red line is a fitting curve.). Temperature dependence of (**c**) Seebeck coefficient and (**d**) Thermal conductivity for Ru_3_Sn_7_ single crystal in zero field.

The temperature dependence of the electrical resistivity of the Ru_3_Sn_7_ single crystal, measured along the <001> crystallographic direction, is presented in Figure 2a. At low temperatures, the resistivity ρ(T) exhibits metallic behavior, characterized by a decrease in resistivity as the temperature is lowered. The residual resistivity of about 0.13µΩ cm at 2 K, and 6.22 µΩ cm at room temperature. The residual resistivity ratio (RRR), defined as the ratio of the resistivity at 300 K to that at 2 K (ρ (300 K)/ρ (2 K)), is calculated to be 46.03. This relatively high RRR value suggests that the Ru_3_Sn_7_ single crystal possesses a reasonably good crystallinity, as a higher RRR typically correlates with fewer defects and impurities in the crystal lattice. The temperature dependence of the resistivity, as depicted in Figure 2a, follows the general trend observed in typical metals, where resistivity decreases with decreasing temperature due to reduced electron scattering. At higher temperatures (above ~25 K), the resistivity is dominated by electron-phonon scattering, which increases linearly with temperature, as expected from the Bloch–Grüneisen theory. And at low temperatures (below ~10 K), it approaches a constant value, corresponding to the residual resistivity. The Ru_3_Sn_7_ resistivity at low temperature is well fitted by the formula ρ = ρ_0_ + AT^2^ (A = 0.00311 μΩcm/K^2^) (ρ_0_ and A represent the residual resistivity and T^2^-term coefficient of the resistivity, respectively). The T^2^ dependence below ~25 K signifies dominant electron-electron scattering, contrasting with conventional phonon-scattering metals. The exceptionally high RRR suggests carrier mobility. This combination of significant electronic correlations and high mobility establishes the foundation for observing quantum oscillations.

As illustrated in Figure 2b, the temperature-dependent isobaric specific heat, denoted as C_P_(T), was systematically measured across a comprehensive temperature range spanning from 2 K to 150 K. The measurements were conducted on a carefully selected single crystal, with a mass of 17.9 mg. The heat capacity data obtained from these single crystals were analyzed under zero magnetic field conditions and were fitted to the theoretical model described by the equation C/T = γ + βT^2^. Through this fitting procedure, the electronic specific heat coefficient, γ, was determined to be 20.32 mJ mol^−1^ K^−2^, substantially larger than ordinary metals (e.g., Cu: ~0.7 mJ mol^−1^ K^−2^). This enhancement likely arises from the flat bands near Dirac points or electronic correlations, consistent with predicted topological features.

Additionally, the Seebeck coefficient, denoted as *S*, and the corresponding thermal conductivity data are comprehensively presented in Figure 2c,d across the measured temperature range. *S* is a fundamental parameter in the study of thermoelectric effects, defined as the ratio of the induced electromotive force per unit length to the applied negative temperature gradient. Mathematically, the Seebeck coefficient for a conductive material can be expressed as $S=-\frac{∆V}{∆T}$, where Δ*V* represents the voltage difference and Δ*T* is the temperature gradient. In this study, the Seebeck coefficient measurements were conducted on high-quality single crystalline Ru_3_Sn_7_ samples using the Physical Property Measurement System (PPMS) equipped with a 9 Tesla magnetic field. The experimental results reveal two distinct sign reversals in the Seebeck coefficient, occurring at 15 K and 60 K. *S* exhibits a pronounced minimum at approximately 25 K, which intriguingly coincides with the maximum value of thermal conductivity, suggesting a potential correlation between these two physical quantities. There is a close physical connection between the Lifshitz transition and the sign change of the Seebeck coefficient, and this correlation mainly originates from the sensitivity of both to the changes in the electronic structure of materials. which may imply a Lifshitz transition in Ru_3_Sn_7_.

**Figure 3:**
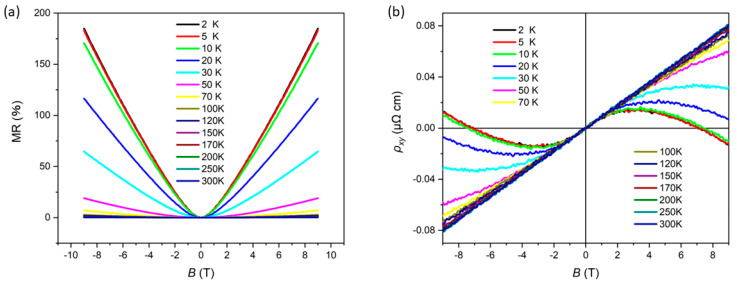
(**a**) Lines provide the MR in the ±9 T region, including data measured at various temperatures. (**b**) Hall resistivity ρ_yx_.

The magnetoresistance (MR), defined as MR = [ρ_xx_ (B) − ρ_xx_ (0)]/ρ_xx_ (0), was systematically measured at various temperatures, and the results are presented in Figure 3a. To gain deeper insights into the multiband electronic structure of the material, the Hall resistivity (*ρ_xy_*) was also investigated across a range of temperatures, as illustrated in Figure 3b. At temperatures above 50 K, the Hall resistivity exhibits a linear dependence on the magnetic field *B* with a positive slope, indicating that hole carriers dominate the transport properties in this regime. The nonlinear behavior of *ρ_xy_* observed at lower temperatures may effectively describe the contributions of both electron and hole carriers. These results are consistent with the Seebeck coefficient data, revealing competition between electron and hole carriers at low temperatures. Notably, at temperatures above 50 K, the Hall curves confirm the predominance of hole carriers, which aligns with the observation that the Seebeck coefficient remains positive in this temperature range.

These phenomena are quantitatively explained by the two-band model:

$$\rho_{yx}=\frac{B}{e}\frac{\left(n_{h}{\mu_{h}}^{2}-n_{e}{n_{h}}^{2}\right)+{\left(n_{h}-n_{e})(\mu_{e}\mu_{h}B\right)}^{2}}{{\left(n_{e}\mu_{e}+n_{h}\mu_{h}\right)}^{2}+{\left(n_{h}-n_{e}\right)}^{2}(\mu_{e}\mu_{h}B)}$$

where n_e_, n_h_, and μ_e_, μ_h_ are carrier densities and mobilities. The high-*T* hole dominance (n_h_ > n_e_) shifts to carrier compensation at low *T*, causing nonlinear ρ_yx_. The sign reversals in *S* reflect competition between electron/hole contributions to thermopower, with extrema corresponding to optimal compensation (n_h_ ≈ n_e_)

**Figure 4:**
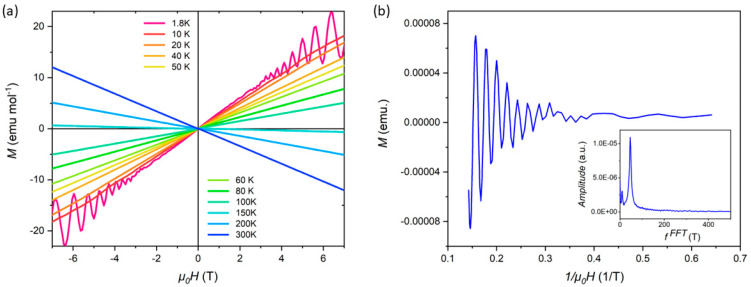
(**a**) *M(H)* at different temperatures. (**b**) Fourier transform (FFT) spectra of the oscillations measured at 2 K. The illustration shows the frequency extracted from FFT analysis.

Finally, in order to gain deeper insights into the electronic structure and Fermi surface properties of the material, we conducted detailed measurements of the quantum oscillations in the isothermal magnetization, *M*(*H*), under applied magnetic fields of up to 7 T. Quantum oscillations (2 K), also known as de Haas-van Alphen (dHvA) oscillations, are a powerful tool for probing the Fermi surface topology and the effective mass of charge carriers in crystalline materials. The presence of well-defined oscillations in the magnetization data, as depicted in Figure 4a, strongly indicates that the studied crystal is highly homogeneous and of excellent quality, with minimal impurities or defects that could otherwise dampen or obscure such oscillations.

**Figure 5:**
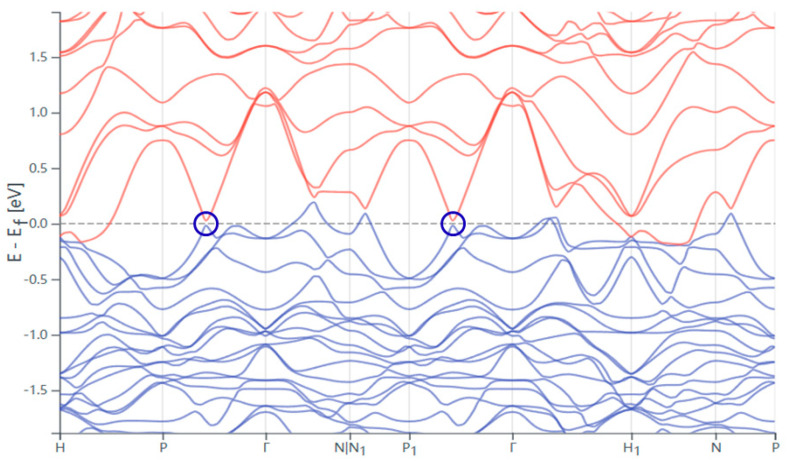
Band structure of Ru_3_Sn_7_ single crystal. (The blue circles are Dirac points). (Topological Material Database. Bilbao Crystallographic Server) [15,16,17].

To further analyze the oscillation data, we performed a Fast Fourier Transform (FFT) analysis (in Figure 4b), which revealed a distinct frequency component at F = 46.97. The clear observation of this frequency, along with the absence of additional spurious peaks in the FFT spectrum, further corroborates the high crystalline quality of the sample and provides valuable information about the electronic band structure and Fermi surface geometry. First-principles calculations (Figure 5, [10,11,12,13,14]) show linearly dispersing bands crossing at Dirac points (arrows) in the bulk Brillouin zone, identifying Ru_3_Sn_7_ as a Dirac semimetal. The observed oscillation frequency matches theoretical Fermi pockets near Dirac nodes. The temperature-dependent oscillation amplitude yields an effective mass extracted from Lifshitz–Kosevich fits: m ≈ 0.25 me, significantly lighter than conventional metals (e.g., Cu: ~1.3 me)—a hallmark of Dirac fermions. When the temperature changes, the electronic states near the Fermi surface may be altered, which in turn can lead to changes in the properties and behavior of charge carriers. As a result, the magnetic susceptibility oscillates, which is a typical feature of topological materials. These findings are consistent with the material’s expected electronic properties and lay the groundwork for further theoretical and experimental investigations into its quantum mechanical behavior.

Notably, the susceptibility anomaly near 15 K (Figure 4a) aligns with the *S*-sign reversal. We attribute this to a temperature-induced Lifshitz transition: Chemical potential shifts relative to Dirac points reconstruct the Fermi surface topology (e.g., opening/closing of electron pockets), modifying carrier balance. This unified mechanism explains correlated transport and magnetic anomalies.

## 4. Conclusions

In summary, we successfully synthesized millimeter-sized single crystals of Ru_3_Sn_7_ and investigated comprehensive measurements, including electrical resistivity, Hall effect, Seebeck coefficient, and thermal conductivity over a wide temperature range from 2 K to 300 K. Our electrical findings and the Seebeck coefficient data suggest that high-quality Ru_3_Sn_7_ single crystals align well with the proposed two-band model. We report the first observation of quantum oscillations in Ru_3_Sn_7_, confirming Dirac points in its electronic structure. Transport data validate an intrinsically compensated two-band system, while Seebeck reversals and magnetic anomalies indicate temperature-driven electronic reconstruction. Combined with band calculations, Ru_3_Sn_7_ is established as a topological Dirac semimetal. Looking forward, the combination of high crystallinity and Dirac fermion physics positions Ru_3_Sn_7_ as a promising platform for exploring quantum transport in compensated systems. Future studies could focus on high-magnetic-field measurements to resolve additional oscillation frequencies and pressure-dependent studies to tune the Dirac point separation, paving the way for novel applications in topological quantum computing.

## Data Availability

The original contributions presented in this study are included in the article material. Further inquiries can be directed to the corresponding authors.
